# Associations Between Hearing and Cognitive Abilities From Childhood to Middle Age: The National Child Development Study 1958

**DOI:** 10.1177/23312165211053707

**Published:** 2021-11-08

**Authors:** Judith A Okely, Michael A Akeroyd, Ian J Deary

**Affiliations:** 1 Lothian Birth Cohort Studies, Department of Psychology, 151029 University of Edinburgh, UK; 2 Hearing Sciences, Division of Clinical Neurosciences, School of Medicine, 170718 University of Nottingham, UK

**Keywords:** lifespan, cognitive hearing science, cohort study, longitudinal survey

## Abstract

Previous cross-sectional findings indicate that hearing and cognitive abilities are positively correlated in childhood, adulthood, and older age. We used an unusually valuable longitudinal dataset from a single-year birth cohort study, the National Child Development Study 1958, to test how hearing and cognitive abilities relate to one another across the life course from childhood to middle age. Cognitive ability was assessed with a single test of general cognitive ability at age 11 years and again with multiple tests at age 50. Hearing ability was assessed, using a pure tone audiogram, in childhood at ages 11 and 16 and again at age 44. Associations between childhood and middle-age hearing and cognitive abilities were investigated using structural equation modelling. We found that higher cognitive ability was associated with better hearing (indicated by a lower score on the hearing ability variables); this association was apparent in childhood (*r*  =  -0.120, *p* <0.001) and middle age (*r*  =  -0.208, *p* <0.001). There was a reciprocal relationship between hearing and cognitive abilities over time: better hearing in childhood was weakly associated with a higher cognitive ability in middle age (*β*  =  -0.076, *p*  =  0.001), and a higher cognitive ability in childhood was associated with better hearing in middle age (*β*  =  -0.163, *p* <0.001). This latter, stronger effect was mediated by occupational and health variables in adulthood. Our results point to the discovery of a potentially life-long relationship between hearing and cognitive abilities and demonstrate how these variables may influence one another over time.

The number of interdisciplinary studies examining the relationship between auditory and cognitive processes is on the rise. This area of study, sometimes termed Cognitive Hearing Science or Auditory Cognitive Science, fills the explanatory gap between “pure” cognitive and auditory sciences (Arlinger et al., 2009). The link between hearing and cognitive function at early and later life-stages has been a focus of the field: numerous studies report on the potential cognitive developmental challenges faced by children with a hearing impairment and the interaction between declining hearing and cognitive abilities in older adults (e.g. Arlinger et al., 2009; Loughrey et al., 2017; Marschark, 2006; Purcell et al., 2016). There is, however, a shortage of research considering the relationship between hearing and cognitive abilities from a life-course perspective. Here we consider this question, using data from the National Child Development Study 1958 (NCDS; Brown & Goodman, 2014; Power & Elliott, 2006), a cohort study with data on hearing and cognitive abilities in childhood and middle age.

Fluid intelligence (abstract reasoning or the ability to solve unfamiliar problems), processing speed (the time required to process information), visuospatial ability (mental representation and manipulation of visuospatial information), crystallised ability (learned knowledge and experience), and memory are all types of cognitive ability. These domains, although conceptually distinct, tend to be positively associated (McGrew, 2005). Indeed, a key finding in cognitive ability research is that scores on different cognitive ability tests are correlated, regardless of the type of mental ability involved (Deary, 2020). This shared variance between cognitive ability tests can be extracted using factor analytic methods and is termed general cognitive ability or ‘*g’ (*Deary, 2020*;* Spearman, 1904*)*. This measure of general cognitive ability is highly correlated with intelligence quotient (IQ) scores derived from single IQ tests (Jensen, 1992). On average, levels of crystallised ability remain relatively stable in older age but other domains of cognitive function tend to decline from early-to-mid adulthood onwards (Salthouse, 2019). There is also substantial variation between individuals with some experiencing more severe cognitive decline than others. Change in general cognitive ability appears to account for a substantial proportion (around 60%) of between-person differences in cognitive change across different cognitive domains and tests (Ghisletta et al., 2012; Tucker-Drob et al., 2019). Despite the age-related changes described above, the rank order of cognitive differences remains relatively stable throughout life. Long-term follow-up studies have shown that about 50% of the variance in general cognitive ability in older age is explained by levels of general cognitive ability in childhood (Deary et al., 2000; Gow et al., 2011).

Hearing abilities can be assessed on various levels from the simplest auditory detection tasks (e.g. pure-tone audiometry), conceptually more complicated auditory discrimination (e.g. the ability to differentiate between auditory stimuli), to comprehension (e.g. the ability to understand the meaning of speech under various auditory conditions). It is reasonable to assume that abilities at the higher end of this auditory hierarchy, such as comprehension, require greater engagement of top-down processes (linguistic knowledge, working memory, and attention) particularly when auditory conditions are sub-optimal (Stenfelt & Rönnberg, 2009). Research with older adults indicates that performance on more complex comprehension tests is more strongly positively associated with cognitive function than performance on simpler hearing threshold tests (Yuan et al., 2018). Relative to research on cognitive ability, less is known regarding within-person trajectories of hearing abilities across the life course (Russ et al., 2018). Findings from short-term longitudinal studies with adults indicate that hearing thresholds gradually and continuously increase at an average of 3 decibels (dB) per decade before the age of 55 and 9 dB per decade thereafter (Davis et al., 1991; Lee et al., 2005). Earlier work with the longitudinal NCDS dataset has shown that hearing thresholds in childhood (at ages 7, 11, and, 16 years) significantly and positively predict hearing thresholds at age 44 (Ecob, 2008).

Deaf children with appropriate exposure to sign language can follow typical developmental trajectories (Loots et al., 2005; Marschark et al., 2001) and perform similarly to hearing children on tests of non-verbal intelligence (Marschark, 2006; Vernon, 2005/1968). However, it has also been demonstrated that deaf and hearing-impaired children tend to perform less well on certain cognitive tests (Conway et al., 2009; Kral et al., 2016). Several theories have been developed to account for this observation. The authors of the auditory scaffolding hypothesis argue that sound is an inherently temporal signal and that the absence of auditory stimulation, early in life, might therefore slow the development of cognitive abilities that involve the processing of temporal or sequential patterns (Conway et al., 2009). Another model, the auditory connectome model, considers how the brain's connectivity is affected by sensory loss (Kral et al., 2016); it highlights the neural connections between the auditory system and other cortical regions including those supporting higher-level cognitive abilities. It is proposed that changes to these connections, induced by hearing loss, could have downstream consequences for the development of cognitive abilities including sequential processing, concept formation, and executive functions i.e. the capacity to control and coordinate cognitive processes (this label, often applied in neuropsychological research, overlaps with that of fluid cognitive ability, described earlier (Salthouse et al., 2008; Salthouse & Davis, 2006)).

Other studies of children with unilateral hearing loss or milder forms of hearing impairment (i.e. >15 and <35 dB hearing loss [HL]) show that these conditions, which can remain undetected and untreated (Matkin & Wilcox, 1999), may also be related to lower academic achievement and performance on IQ tests (Purcell et al., 2016; Tharpe et al., 2009). Although, others report that children with a mild hearing impairment perform similarly (in terms of language, reading, and behaviour) to their normally hearing peers (Wake et al., 2006). Understanding the relationship between hearing loss and cognitive development is complicated by the heterogeneity of hearing-impaired and deaf populations (Marschark, 2006): epidemiological data from the UK indicates that 27% of hearing impaired children have other disabilities, and that 10% have a syndromic condition (Fortnum et al., 2002). Thus, aassociations between hearing impairment and cognitive development could, in some cases, result from an etiology that both outcomes share (Purcell et al., 2016).

The relationship between hearing and cognitive abilities has also been studied in populations without hearing impairment, particularly in studies of intelligence differences. Some studies in this context investigate the nature of intelligence—as measured using psychometric tests—and its relation to sensory function. Some of the earliest empirical investigations into intelligence adopted this approach. Spearman (1904), building on Galton's (1883) theory of a functional link between sensory and cognitive abilities, found a strong correlation between general sensory discrimination and general cognitive ability in children (Spearman, 1904). Deary (1994b) provided a detailed description, critique, and re-analysis of studies examining cognitive function and sensory discrimination (including auditory) studies between 1904 and 1917. He found that there generally was a small, significant positive association between higher cognitive ability and better sensory discrimination in these early studies. More recently, studies testing information processing models of intelligence have re-examined the link between intelligence and the senses. Studies with adult or child participants have documented associations between higher intelligence test scores and better auditory processing including processing speed, discrimination, and acuity (Deary et al., 1989b; Deary et al., 2004; Helmbold et al., 2006; McCrory & Cooper, 2005; Parker et al., 1999; Raz et al., 1987; Watson, 1991), with reported correlations generally ranging between *r*  =  0.3 and *r*  =  0.6 (though these were sometimes associations between latent traits and not correlations between two single variables). Further investigations have compared different forms of auditory processing and their relation to cognitive function. For instance, Deary (1994a) found that auditory processing speed was more strongly related than pitch discrimination to cognitive ability. There is also evidence that auditory and visual processing speed are correlated and that both measures are associated with cognitive function (Deary et al., 1989a).

It is still unclear why cognitive and hearing abilities are positively associated in normal hearing populations. There are at least three, non-exclusive, accounts of this association in the literature. The first, briefly described above, posits that speed of sensory processing is causally related to cognitive development; perhaps faster processing speed confers a cognitive developmental advantage (Tallal et al., 1993). In support of this idea, a study using cross-lagged panel data of auditory processing speed and cognitive ability in children, found that auditory processing speed at age 11 accounted for around 6% of the variance in subsequent cognitive ability, assessed at age 13 (Deary, 1995). A second view is that sensory abilities are a consequence rather than a cause of cognitive ability, the idea being that a higher cognitive ability can support more efficient processing of sensory information (e.g., Ceci, 1990). Thirdly, the correlation between cognitive and hearing abilities is consistent with the “system integrity” hypothesis, that there is an underlying trait of “optimal bodily functioning” which originates early in life and accounts for shared variance in different mental and physical functions (Deary, 2012). From this perspective, hearing and cognitive abilities may not be causally related; rather, both processes are dependent on overall bodily functioning.

Research into the relationship between hearing and cognitive abilities in childhood is paralleled by work, on the same topic, with older adults. The volume of research in this latter area has grown rapidly following the suggestion that hearing impairment might represent a potentially modifiable risk factor for age-related cognitive decline and dementia (Livingston et al., 2017; Loughrey et al., 2017). In a recent meta-analysis of 40 observational studies, Loughrey et al. (2017) found a small but significant correlation between age-related hearing impairment and poorer cognitive function, cognitive impairment and dementia risk. However, the mechanisms underlying the relationship between hearing and cognitive abilities in older age are still unclear. One theory positing a causal link between these abilities, the “sensory deprivation” hypothesis, suggests that hearing impairment negatively impacts cognitive function by reducing access to intellectual stimulation (Lindenberger & Baltes, 1994). Another view put forward by the “effortfulness” or “information degradation” hypothesis is that, in people with a hearing impairment, cognitive resources are diverted to the processing of auditory information resulting in poorer performance on other cognitive tasks (Lindenberger & Baltes, 1994; McCoy et al., 2005). The “cognitive load on perception” hypothesis views this association from the opposite direction and suggests that reduced cognitive ability negatively impacts auditory processing, particularly in the context of more complex tasks such as speech-in-noise hearing. From this perspective, declines in cognitive ability should precede declines in auditory abilities (Lindenberger & Baltes, 1994; Pronk et al., 2019). It is also possible that cognitive and hearing abilities in older age are both influenced by a third factor (Lindenberger & Baltes, 1994; Tobias et al., 1988). This suggestion is consistent with the “common cause” hypothesis, that a common physiological ageing process drives declines in basic sensory and cognitive functions (Baltes & Lindenberger, 1997; Christensen et al., 2001). This model of ageing predicts that measurements of the relationship between sensory and cognitive functions will give higher correlations in later life as age-related physiological declines begin to impact both sensory and cognitive processes.

The theories outlined above describe changes in cognitive and hearing abilities that are often considered specific to older age. However, as described earlier, hearing threshold levels and certain cognitive abilities begin to decline in midlife. Several studies have tested models of ageing, cognition, and auditory processing with samples of middle-aged adults (Gallacher et al., 2012; Humes, 2015; Merten et al., 2020; Moore et al., 2014; Schubert et al., 2017). Overall, these studies confirm that declines in sensory and cognitive processing are apparent in midlife – albeit to a lesser degree than in older age – and that hearing and cognitive abilities tend to be positively correlated at this life stage (although some authors report only weak associations, see Merten et al., 2020).

Research with children, middle-aged and older adults points to a positive association between hearing and cognitive abilities at multiple stages of the life course. Theories regarding the nature of these associations in childhood and adulthood have largely developed independently from one another and focus on processes specific to those life stages i.e. development in childhood and ageing processes in adulthood and older age. The association between hearing and cognitive abilities has rarely been viewed from a life-course developmental perspective, that is, how hearing and cognitive abilities in early life might relate to the relationship between those same variables at an older age in the same sample.

There are multiple mechanisms by which hearing and cognitive abilities could relate to one another across the life course. Firstly, it is possible that associations between hearing and cognitive abilities, established early in life (via developmental processes or reflecting a “system integrity” effect) are tracked over time and are therefore also present at later life stages. This possibility, which emphasises the stability of individual differences in hearing and cognitive abilities, contrasts with the prediction made by the common cause hypothesis (Baltes & Lindenberger, 1997), which predicts that associations between hearing and cognitive abilities will emerge or become stronger in older age. Secondly, childhood cognitive ability could contribute to the risk of hearing loss in adulthood, potentially via its positive association with health literacy (Murray et al., 2011) and relevant health behaviours, such as lower rates of smoking (Wraw et al., 2018), and lower risk of chronic diseases (Batty et al., 2007; Singh-Manoux et al., 2009) including those associated with hearing loss (Fowler & Jones, 1999; Gates et al., 1993; Gopinath et al., 2010; Nomura et al., 2005). We found support for this direction of effect in our previous observational study, using data from the Lothian Birth Cohort 1936; in that study, a higher cognitive ability in childhood was related to a lower risk of hearing impairment at age 76 (Okely et al., 2019). The opposite direction of effect, from childhood hearing ability to adult cognitive ability is also plausible, although less well documented. For instance, the reported positive association between childhood hearing ability and childhood cognitive ability and academic achievement could determine access to subsequent experiences that support cognitive development or maintenance in adulthood such as university education (Clouston et al., 2012) and occupational complexity (Smart et al., 2014). Figure 1 summarises some potential mechanisms linking hearing and cognitive abilities in childhood and adulthood, and potential mechanisms, proposed in this paper, linking these variables across the life course.

**Figure 1.**
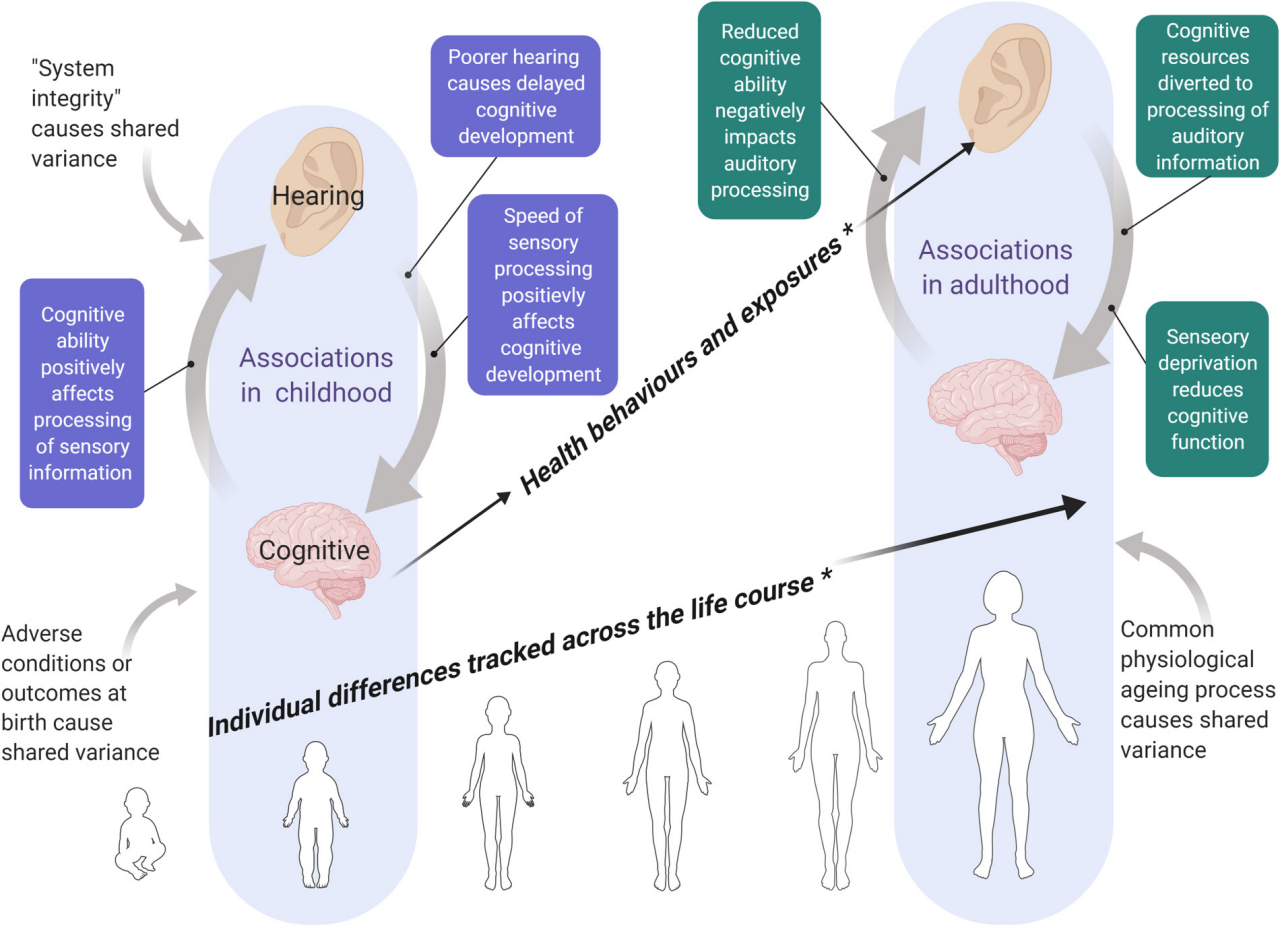
*Summary of theories accounting for associations between hearing and cognitive abilities in childhood and in adulthood*. *Note*. Pathways marked with a * represent potential mechanisms linking hearing and cognitive abilities across the life course, these pathways are proposed and tested in this report. The figure shows some of the common theories accounting for associations between hearing and cognitive abilities in childhood and adulthood but is not an exhaustive list. Figure created with BioRender.com.

In the present study we took advantage of a unique research opportunity to demonstrate that there is a life-long – from childhood to middle age – reciprocal and dynamic relationship between hearing and cognitive abilities. The NCDS is a longitudinal cohort study of individuals living in Scotland, England, and Wales who were born during one week in 1958. It includes assessments of hearing and cognitive abilities in childhood and in middle age. We note here that, because the sample was drawn from the general population, the proportion of participants with mild to severe hearing loss is low and therefore, specific mechanisms linking hearing loss with cognitive ability could not be tested. Nevertheless, participants show significant variance in hearing threshold levels in childhood and adulthood, albeit predominantly within the normal hearing range. Using these data, we firstly tested the “tracking hypothesis” that is, whether associations between hearing and cognitive abilities in childhood are tracked over time and therefore account for associations between those same variables in middle age. Secondly, we tested for so-called “cross-lagged” effects (Newsom, 2015): from childhood cognitive ability to middle-age hearing ability, and from childhood hearing ability to middle-age cognitive ability. Thirdly, we tested whether any such cross-lagged effects were mediated by an extensive set of occupational, demographic, lifestyle and health variables. In this final step, we focused on the cross-lagged effect from childhood cognitive ability to middle-age hearing ability as there is evidence from previous studies that higher cognitive ability predicts health behaviours and exposures associated with auditory health.

## Methods

### Participants

The NCDS is a longitudinal study of people living in Scotland, England, and Wales who were born in a single week in March 1958 (Brown & Goodman, 2014; Power & Elliott, 2006). The study began as the British perinatal mortality survey. It included 17,415 participants at birth, when data from medical records and maternal characteristics were collected. Subsequently, cohort members were followed up at ages 7, 11, 16, 23, 33, 42, 44–45, 46, 50, and 55 years. Immigrants with the same birth dates as the original cohort were added to the sample at ages 7, 11, and 16, resulting in a total cohort sample of 18,558. The present study used data from ages 11, 16, 42, 44–45, and 50. Rates of sample attrition are relatively low (Power & Elliott, 2006) but do result in a substantially reduced sample size by age 50 (N  =  9,790). Previous analysis with the 45-year-old sample shows that participants who remained in the study are broadly representative of the original sample at birth and at age 7; however, there is some underrepresentation of disadvantaged and minority groups in the middle-aged sample (Atherton et al., 2008).

Verbal informed consent was sought from respondents or respondents’ parents for each survey, written consent was recorded at the biomedical survey at age 44. Ethical approval was obtained from the South East and London multicenter research ethics committees (REC reference numbers: 01/1/44; 08/H0718/29; 12/LO/2010). See Shepherd (2012) for further details.

### Measures

#### Cognitive Ability

Cognitive ability was assessed at age 11. Participants sat a series of tests at school including a test of general cognitive ability consisting of 40 verbal and 40 non-verbal items devised by the National Foundation for Educational Research in England and Wales. This test was found to correlate strongly (*r*  =  0.93) with an IQ-type test used for secondary school selection (Douglas, 1964). Cognitive ability was assessed again at age 50, this time with four tests designed to assess memory, verbal fluency, and perception and attention (processing speed). Memory was assessed with a word list learning task where participants recall a list of 10 common words immediately and after a delay. Verbal fluency was assessed using a task where participants name as many different animals as possible in one minute. Processing speed was assessed by a letter cancelation task where participants are given a page of random letters and are instructed to cross out as many ‘Ps’ and ‘Ws’ as possible in one minute. Cognitive tests at age 50 were conducted at the participant's home as part of a computer assisted personal interview (Bhamra et al., 2010; Matthew Brown & Dodgeon, 2010). We created a latent variable representing general cognitive ability at age 50 using performance on the tests of verbal fluency, memory, and processing speed as indicators.

#### Hearing Ability

Hearing was measured at ages 7, 11, 16, and 44–45 (henceforth 44) years using a pure tone audiogram (performed by air conduction) in each ear. This method measures hearing threshold levels for a range of frequencies. At ages 7, 11, and 16 the audiogram was conducted in locally available audiometer facilities and included frequencies of 0.25, 0.5, 1, 2, 4, and 8 kHz. We created a latent variable representing childhood hearing ability using hearing threshold levels at ages 11 and 16. Because hearing losses are typically smaller and therefore harder to detect at low frequencies (e.g. Akeroyd et al., 2019; Davis, 1995) we did not include hearing threshold levels for 0.25 and 0.5 kHz at either age. At age 44, a shorter audiogram, including only 1 and 4 kHz, was conducted in the participant's home by a trained study research nurse (Ecob, 2008). We created a latent variable representing middle-age hearing ability using hearing thresholds at 1 and 4 kHz. Because higher scores on the latent hearing ability variables represent poorer hearing, we will refer to these variables as childhood and middle-age hearing threshold.

Table 1 provides a summary of the hearing and cognitive ability variables including the timing, method, and location of auditory and cognitive assessments.

**Table 1.** Summary of the Hearing and Cognitive Abilities Variables: Timing, Method, and Location of Assessments.

Domain	Age	Test	Location
Cognitive ability - childhood	11 years	General cognitive ability test	School
Cognitive ability - middle age	50 years	Memory, verbal fluency, and processing speed tests	Home
Hearing - childhood	11 years	Audiogram at octave frequencies 1-8 kHz	Locally available audiometer facilities
Hearing - childhood	16 years	Audiogram at octave frequencies 1-8 kHz	Locally available audiometer facilities
Hearing - middle age	44 years	Hearing thresholds at 1 and 4 kHz	Home

#### Covariates

We identified covariate variables that might confound the association between hearing and cognitive abilities in childhood, or potentially mediate the association between childhood cognitive ability and middle-age hearing ability. Potentially confounding variables included sex, history of middle ear dysfunction, and childhood social class. Results from an otoscopic examination of each ear at age 11 were used as a proxy measure of middle ear dysfunction. As has been done previously (Ecob et al., 2011) participants with “inflamed”, “scarred”, or “abnormal-other” results were categorized as having a history of middle ear dysfunction. Childhood social class was based on father's occupation at the participant's birth, or if not available at birth, at age 7. Occupations were grouped into six categories: professional (I), managerial/technical (II), other non-manual (IIInm), skilled manual (IIIm), partly skilled (IV) and unskilled manual (V). Potentially mediating variables included occupational noise exposure, adult occupational social class, physical activity, smoking status, alcohol consumption, history of diabetes, BMI, and systolic blood pressure. Most potentially mediating variables were recorded at age 42. Adult occupational social class was based on the participant's current occupation. As with childhood social class, participant occupations were grouped into categories of professional (I), managerial/technical (II), other non-manual (IIInm), skilled manual (IIIm), partly skilled (IV) and unskilled manual (V). Participants indicated whether they did any regular exercise and, if so, how often. This information was used to create a variable with seven categories ranging from no exercise to exercise every day. Participants reported whether they were a “never smoker”, “occasional or ex-smoker”, or “a current smoker”. Participants were asked to report how often they drank alcohol of any kind. The seven response options ranged from “never had an alcoholic drink” to “on most days”. Participants were also asked to report whether they had or ever been told that they had diabetes. Additional potentially mediating variables were taken from the biomedical survey at age 44. These measures were taken by a trained study research nurse at the participant's home and included a measure of systolic blood pressure (which was the mean of three readings) and BMI (calculated using a measure of standing height to the nearest millimeter and weight in light clothing to the nearest 0.1 kg). Occupational noise exposure was assessed at age 44 with the self-report question “Have you ever worked in a place that was so noisy that you had to shout to be heard?” Response options were “no, never”; “yes, for less than 1 year”; “yes, for 1–5 years”, and “yes, for over 5 years”.

Distance visual acuity was recorded at age 11 using a conventional Snellen chart at 6.1 m, and at age 44 using a LogMAR crowded test at 1.5 m (results were converted to the Snellen equivalent). Using best achieved distance visual acuity in each eye (corrected if prescribed) and a cut-off applied previously with this cohort (Bountziouka et al., 2017), we categorized participants as having either normal vision (6/4 to 6/9.5 in both eyes) or a visual impairment (6/12  +  in either eye) at age 11 and at age 44. These variables were not included in the main analysis; however, we did test whether participants with a visual impairment, at age 11 or 44, had significantly higher hearing threshold levels at the same age.

### Analytical Sample

**Figure 2.**
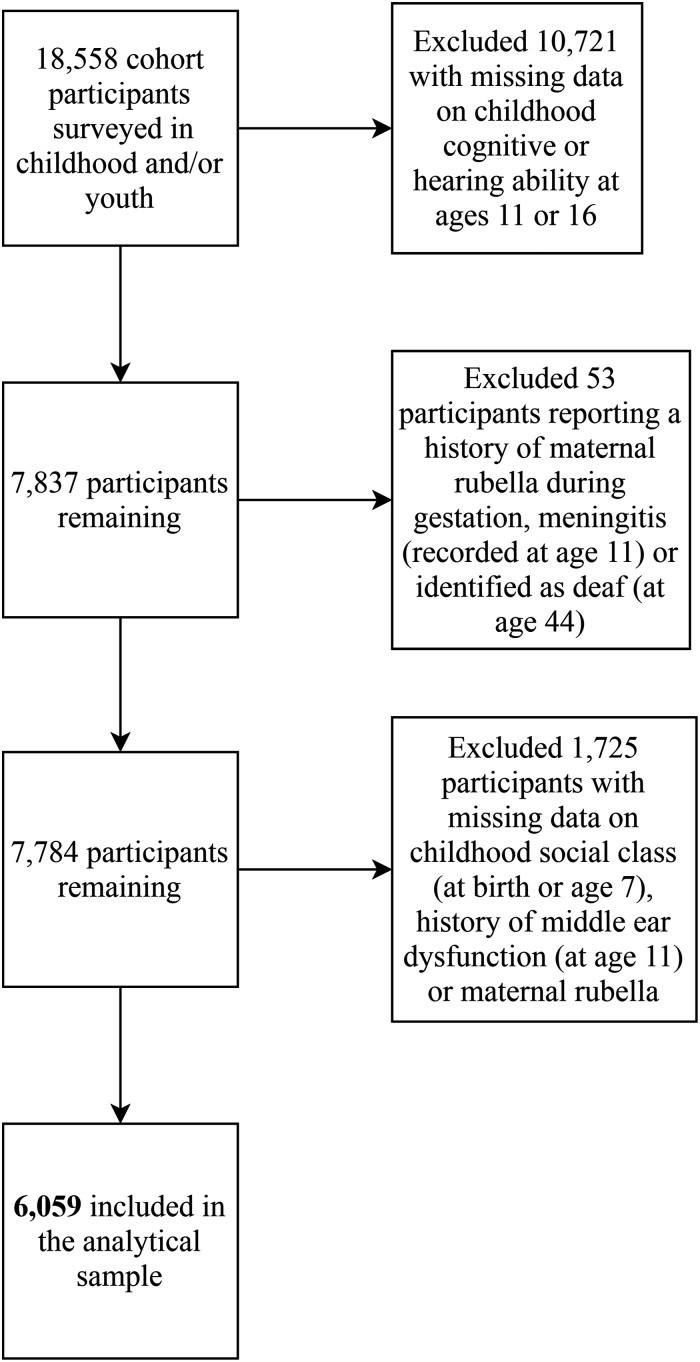
Flow chart showing participants excluded from the analytical sample.

Owing to the long-running nature of the NCDS, there are missing data on some variables. We excluded participants from the analytical sample if they had missing data on the hearing or cognitive ability variables at ages 11 or 16. In order to reduce the potential effect of profound hearing loss on our results, we excluded participants who were identified as deaf at age 44, or had a history of illness associated with hearing loss (history of meningitis reported at age 11, or maternal rubella during gestation). We further excluded participants with missing data on independent (exogenous) variables; these were sex, history of middle ear dysfunction, and childhood social class (as these participants would be automatically excluded from the final model which was run using weighted least squares mean and variance adjusted estimation [WLSMV]). Participants with missing data on history of meningitis or maternal rubella were also excluded. These exclusions resulted in an analytical sample of 6,059 participants. The flow chart in Figure 2 shows the number of participants excluded from the analytical sample for each of the steps described above. Supplementary Tables 1–3 show the characteristics of participants included and excluded from the analysis and the number of participants with missing data on each variable in the study. Excluded participants generally had a higher hearing threshold level, performed less well on most cognitive tests, consumed less alcohol, were more likely to have a father with a more manual occupation, were more likely to smoke, and were more likely to report greater occupational noise exposure.

### Analysis

**Figure 3.**
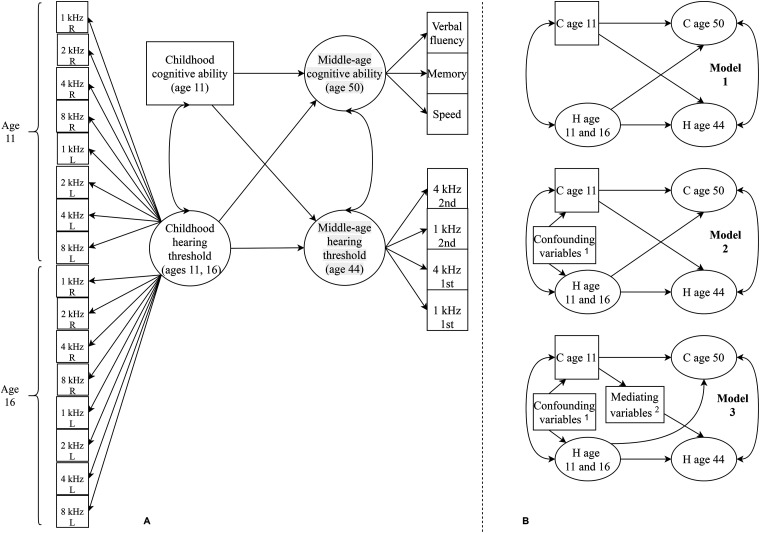
Summary of the structural equation model testing for associations between cognitive and hearing abilities including the indicators of each latent variable in the model (panel A) and simplified diagrams of models 1, 2, and 3 (panel B). Note. C  =  cognitive ability, H  =  hearing threshold. Double headed arrows represent correlations and single headed arrows represent regression effects. Squares and rectangles represent observed variables, ellipses represent latent variables. ^1^Confounding variables were sex, history of middle ear dysfunction, and childhood social class. ^2^Mediating variables were occupational noise exposure, adult occupational social class, physical activity, smoking status, alcohol consumption, history of diabetes, BMI, and systolic blood pressure.

We examined the association between childhood and middle-age hearing and cognitive abilities using structural equation modelling (SEM). An advantage of SEM is that it is possible to simultaneously model manifest and error-free latent variables and the potential relationships between them. Model development progressed in two stages. We firstly ran two preliminary cross-sectional models: one of childhood hearing threshold and cognitive ability and another of middle-age hearing threshold and cognitive ability. This approached allowed us to test whether the hearing and cognitive constructs could be modelled as latent variables and to test for cross-sectional associations between them. We used hearing threshold levels of left and right ears at ages 11 and 16 as indicators of overall childhood hearing threshold. We used hearing thresholds at 1 and 4 kHz for each ear as indicators of overall hearing threshold at age 44. Cognitive ability at age 50 was modelled using three indicators of verbal fluency, memory, and processing speed. We summed scores on the immediate and delayed recall tests to create a single indicator of memory and used total number of letters scanned on the letter cancelation test as an indicator of processing speed. Following this first step, we ran three main longitudinal models estimating associations between childhood and middle age hearing thresholds and cognitive abilities. These models are summarized in Figure 3. The first model (Model 1) specified correlations between hearing thresholds and cognitive abilities in childhood and in middle age (so representing associations between these variables at each life stage), regression paths from childhood cognitive ability to middle-age cognitive ability and from childhood hearing threshold to middle-age hearing threshold, and cross-lagged effects from childhood cognitive ability to middle-age hearing threshold and from childhood hearing threshold to middle-age cognitive ability. A second model (Model 2) additionally controlled for the potentially confounding effects of sex, childhood social class, and middle ear dysfunction. Childhood social class was dummy coded with “professional occupation” as the reference category. A final model (Model 3) additionally controlled for the potentially mediating effects of occupational noise exposure, adult occupational social class, physical activity, smoking status, alcohol consumption, history of diabetes, BMI, and systolic blood pressure. While our main focus in this last step was on the cross-lagged effect from childhood cognitive ability to middle-age hearing threshold, in subsidiary analysis we additionally tested whether any of these variables mediated the association between childhood hearing threshold and middle-age cognitive ability.

All models apart from Model 3 (controlling for potential mediators) were estimated using maximum likelihood (ML) estimation. For Model 3, WLSMV estimation was used, as otherwise it was too computationally demanding for ML estimation owing to the multiple categorical mediators in this model. With WLSMV estimation, categorical mediators are modelled as continuous latent response variables and path estimates are modelled using linear regression (Muthén & Asparouhov, 2015). Models 2 and 3 involved a high number of significance tests; we therefore corrected *p*-values from these models for multiple comparisons using the Benjamini–Hochberg False Discovery Rate (FDR) correction (Benjamini & Hochberg, 1995). P-values of <0.001 were entered as 0.001 for the purposes of the FDR correction. Model fit was assessed using the comparative fit index (CFI), Tucker-Lewis index (TLI), and root-mean-square error of approximation (RMSEA) (Cangur & Ercan, 2015). Following the recommendations of Schermelleh-Engel et al. (2003) we considered model fit values of CFI and TLI ≥ 0.95 and RMSEA ≤ 0.08 as indicators of acceptable fit. Parameter estimates are reported as correlations or standardized betas – which can be interpreted in a similar way to correlations (Acock, 2014): *β* <0.2 is considered a small effect, *β* >0.2 and <0.5 a moderate effect, and *β* >0.5 a large effect. Data preparation, management, plotting, and calculation of descriptive statistics was conducted in the R software environment, version 3.6.1 (R Core Team, 2019) with the aid of R packages dplyr (Wickham et al., 2019), tidyr (Wickham & Henry, 2019), ggplot2 (Wickham, 2016), arsenal (Heinzen et al., 2019), and MplusAutomation (Hallquist & Wiley, 2018). All models were estimated in Mplus, version 8.4 (Muthen & Muthen, 2017).

## Results

Tables 2 and 3 show the characteristics of participants in the analytical sample. In childhood, hearing threshold levels were highest (i.e. poorest) at 1 kHz and lowest at 4 kHz, this pattern was reversed at age 44. The percentage of participants with hearing loss (>25 dB) at any frequency ranged between 0.50 and 1.9% in childhood and between 1.8 and 6.2% in adulthood. Supplementary Tables 4 and 5 report the correlations between indicators of the latent variables of childhood hearing threshold, middle-age hearing threshold, and middle-age cognitive ability. There was a strong correlation between hearing thresholds for different frequencies at age 11 (mean [M] of correlations  =  0.69; range [R] of correlations  *=*  0.57, 0.80) and at age 16 (M  =  0.66; R  =  0.54, 0.75). Correlations between hearing thresholds at different ages (same or different frequencies) were moderate in effect size (M  =  0.26; R  =  0.19, 0.34). Performance on the general cognitive ability test at age 11 was significantly negatively correlated with hearing thresholds at each frequency and age; the effect sizes of these correlations was small (M  =  -0.08; R  =  -0.10 −0.07).

**Table 2.** Summary Statistics of the Hearing and Cognitive Ability Variables.

	Age	Overall (6,059) Mean (SD)	N (%) with hearing loss (>25 dB)	N missing
Hearing threshold at 1 kHz	11	8.46 (7.00) dB	51 (0.84)	0
Hearing threshold at 2 kHz	11	5.88 (6.57) dB	30 (0.50)	0
Hearing threshold at 4 kHz	11	5.68 (6.75) dB	39 (0.64)	0
Hearing threshold at 8 kHz	11	6.73 (7.52) dB	69 (1.14)	0
Hearing threshold at 1 kHz	16	9.69 (7.31) dB	63 (1.04)	0
Hearing threshold at 2 kHz	16	6.04 (7.59) dB	39 (0.64)	0
Hearing threshold at 4 kHz	16	6.07 (8.00) dB	54 (0.89)	0
Hearing threshold at 8 kHz	16	7.75 (8.79) dB	112 (1.85)	0
General cognitive ability test	11	44.63 (15.67)		0
Hearing threshold at 1 kHz	44	6.05 (8.08) dB	68 (1.76)	2,198
Hearing threshold at 4 kHz	44	7.83 (11.86) dB	240 (6.22)	2,203
Number of words correctly recalled	50	6.600 (1.47)		2,094
Number of animals mentioned	50	22.56 (6.29)		2,094
Letter cancellation speed score	50	334.61 (88.55)		2,168
Number of words recalled after delay	50	5.47 (1.82)		2,117

*Note*. Percentage with hearing loss is based on N available for each hearing threshold. Both ears were assessed in childhood and at age 44. For brevity, the table only shows hearing thresholds for each frequency from the best hearing ear for that frequency. kHz  =  kilohertz.

**Table 3.** Summary of Covariate Variables in the Analytical Sample.

Variable	Age	Overall (N = 6,059)	N missing
Sex (female)		2863 (47.3%)	
Childhood social class	0/7		
- unskilled		281 (4.6%)	
- partly skilled		853 (14.1%)	
- skilled manual		615 (10.2%)	
- skilled non-manual		3,021 (49.9%)	
- managerial-technical		770 (12.7%)	
- professional		519 (8.6%)	
Middle ear dysfunction (yes)	11	465 (7.7%)	
Diabetes (yes)	42	71 (1.5%)	1,474
Exercise frequency	42		1,476
- never		1,133 (24.7%)	
- less than 2-3 times a month		114 (2.5%)	
- 2-3 times a month		306 (6.7%)	
- once a week		844 (18.4%)	
- 2-3 days a week		1,000 (21.8%)	
- 4-5 days a week		417 (9.1%)	
- every day		769 (16.8%)	
Alcohol frequency	42		1,474
- most days		958 (20.9%)	
- 2-3 times a week		1,551 (33.8%)	
- once a week		840 (18.3%)	
- 2-3 times a month		472 (10.3%)	
- special occasions only		564 (12.3%)	
- never nowadays		149 (3.2%)	
- never		51 (1.1%)	
Adult social class	42		2,108
- unskilled		231 (5.8%)	
- partly skilled		1,517 (38.4%)	
- skilled manual		808 (20.5%)	
- skilled non-manual		798 (20.2%)	
- managerial-technical		478 (12.1%)	
- professional		119 (3.0%)	
Smoking status	42		1,473
- non-smoker		2,114 (46.1%)	
- ex/occasional smoker		1,369 (29.9%)	
- current smoker		1,103 (24.1%)	
Noise at work	44		2,438
- never		2,526 (69.8%)	
- for less than 1 month		399 (11.0%)	
- for 1-5 years		283 (7.8%)	
- for over 5 years		413 (11.4%)	
BMI	44	27.47 (4.93)	2,227
Blood pressure	44	126.79 (16.29)	2,222

*Note*. Data are presented as Mean (SD) or N (%).

Hearing thresholds at age 44 were significantly positively correlated across ears and frequencies (M  =  0.45; R  =  0.28, 0.61). Scores on the four cognitive ability tests at age 50 were significantly positively correlated with each other (M  =  0.26; R  =  0.09, 0.65). There was a significant negative correlation between most cognitive ability test scores at age 50 and hearing thresholds at age 44 with the exception of letter cancellation speed and hearing thresholds at 1 kHz (M = -0.07; R  =  -0.13, −0.00).

**Figure 4.**
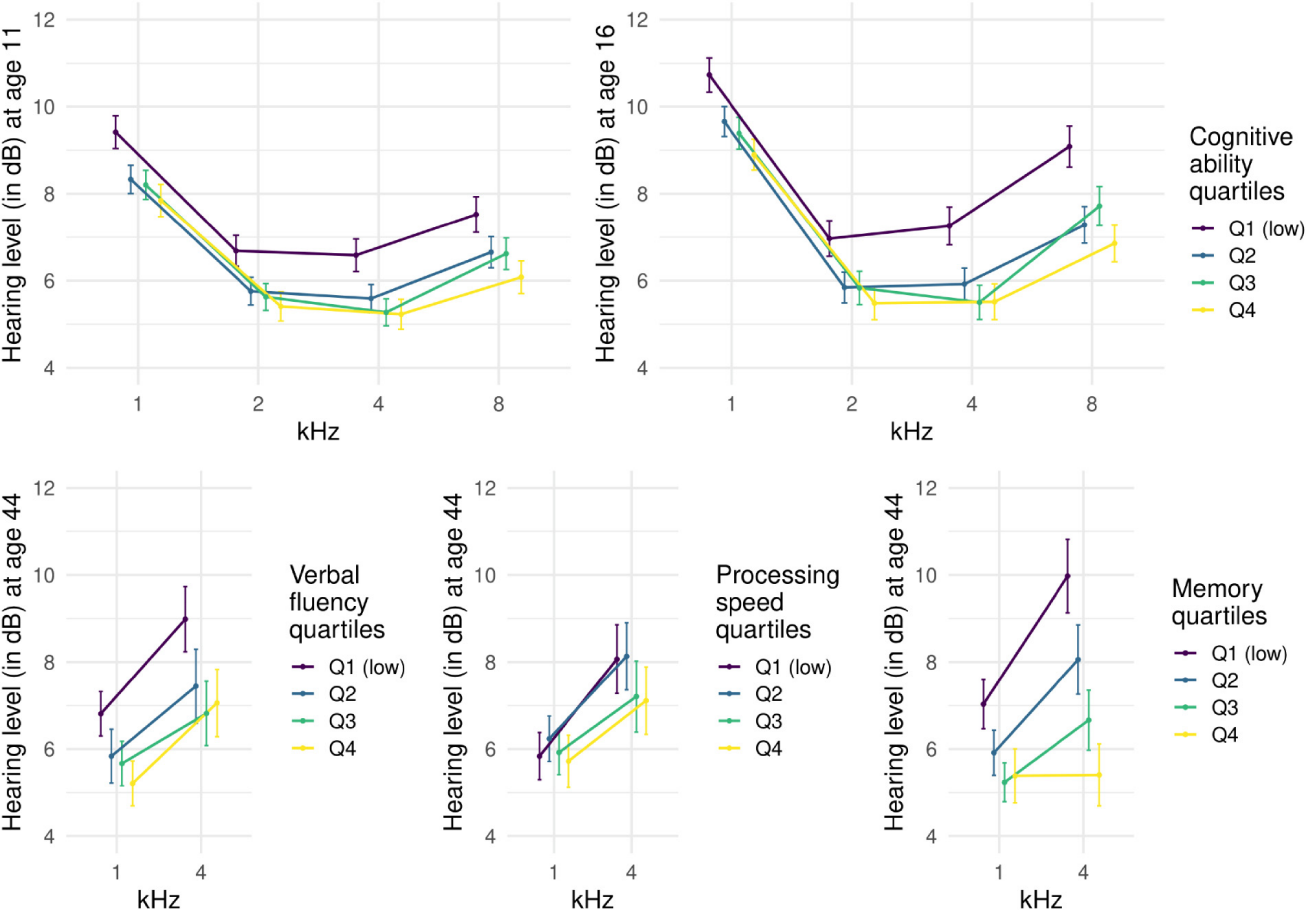
Mean hearing threshold levels at ages 11, 16, and 44 of participants grouped according to quartiles of cognitive ability test scores. Note. Error bars represent 95% confidence intervals. Hearing thresholds for each frequency are taken from the better hearing ear for that frequency. The horizontal positions of the means and confidence intervals have been adjusted to minimise overlap between groups.

The relationship between hearing and cognitive abilities in childhood and middle age is illustrated in Figure 4 using quartiles of cognitive ability scores. The pattern is that hearing is better in those quartiles with the higher cognitive test scores, though with little separation of the middle two cognitive quartiles. The magnitude of the differences between the lowest cognitive ability quartile and the other quartiles is typically between 1–2 dB at the higher frequencies (with the exception of quartiles for memory which show slightly larger differences in hearing thresholds). We emphasise, at this stage, that these effect sizes should not be considered as the associations between hearing and cognitive abilities. To estimate the true magnitude of these associations subsequent analyses combined individual hearing and cognitive variables into latent traits.

Comparisons of participants with normal vision and visual impairment indicated no significant differences in hearing threshold levels at age 11 or 44 (see Supplementary Tables 6 and 7).

### Cross-Sectional Models

We firstly ran a SEM of childhood hearing threshold and cognitive ability (Model 0A). Hearing threshold in childhood was modelled as a single latent factor; hearing thresholds of each ear at ages 11 and 16 (at 1, 2, 4, and 8 kHz) were treated as factor indicators. Cognitive ability in childhood was modelled as an observed variable using the general cognitive ability test score at age 11. We found that the initial model of childhood hearing threshold and cognitive ability, which allowed residual correlations between hearing threshold levels of the same frequency to correlate (between ears and measurement occasions) did not fit the data well: CFI =  0.659, TLI  =  0.512, RMSEA  =  0.206. Poor fit indices are commonly encountered when factors have a high number of indicators (Perry et al., 2015), as was the case here. Modification indices suggested correlations between residuals of hearing thresholds for different frequencies assessed at the same age (11 or 16) for the same and opposing ears. As it was plausible that these sets of indicators would be more closely related, we freed their residual correlations in the model. These modifications resulted in acceptable fit: CFI  =  0.975, TLI  =  0.941, RMSEA  =  0.072. Factor loadings for hearing threshold in childhood were all significant and ranged between 0.324 and 0.813 (these were similar to the loadings in the first more restricted model with fewer correlated residuals). Childhood cognitive ability and childhood hearing threshold were significantly negatively correlated (*r*  =  -0.117, *p* < 0.001) indicating that a higher cognitive ability was associated with a lower overall hearing threshold (i.e. more sensitive hearing).

Next, we ran a model of middle-age hearing threshold and cognitive ability (Model 0B). This model fit the data well: CFI  =  0.996, TLI  =  0.991, RMSEA  =  0.024. Factor loadings for cognitive ability in middle age were significant and ranged between 0.216 and 0.536. Factor loadings for hearing threshold in middle age were also significant and ranged between 0.508 and 0.483. Residuals of hearing thresholds for the same ear or for the same frequency were allowed to correlate. Middle-age cognitive ability and middle-age hearing threshold were significantly correlated (*r*  =  -0.299, *p* < 0.001). Results of these models are shown in Supplementary Figure 1.

### Longitudinal Models

**Figure 5.**
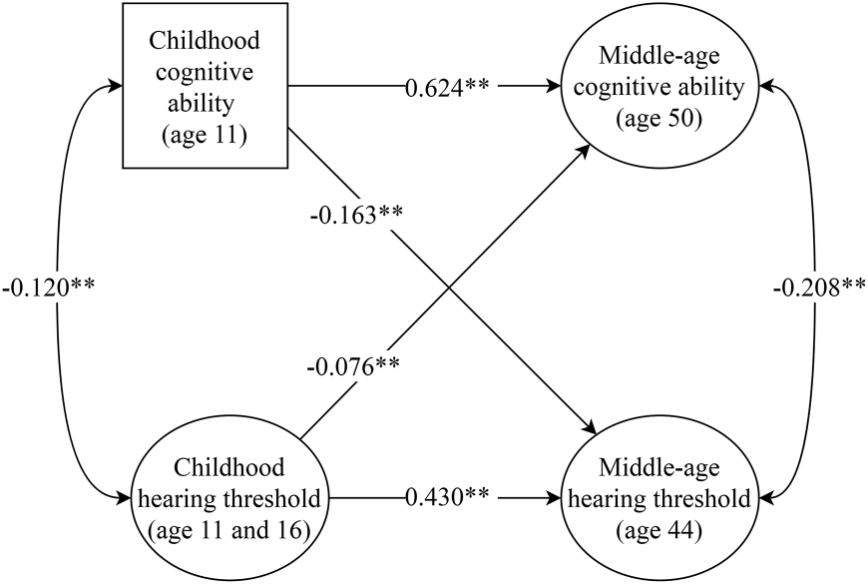
*Standardized parameter estimates from model 1*. *Note*. Double headed arrows represent correlations and single headed arrows represent regression effects. * indicates p < .05. ** indicates p < .01.

Model 1, with no potentially confounding or mediating variables provided an adequate fit to the data CFI  =  0.970, TFI  =  0.955, RMSEA  =  0.046. Estimates from Model 1 are shown in Figure 5 and Supplementary Table 8. There was a significant correlation between hearing threshold and cognitive ability in childhood *r*  =  -0.120, *p* <0.001 and in middle age *r*  =  -0.208, *p* <0.001 such that higher cognitive ability was associated with better hearing (indicated by a lower hearing threshold). Childhood cognitive ability significantly predicted middle-age cognitive ability *β*  =  0.624, *p* <0.001 and childhood hearing threshold significantly predicted middle-age hearing threshold *β*  =  0.430, *p* <0.001, suggesting that these traits were relatively stable from childhood to middle age. The cross-lagged effect from childhood cognitive ability to middle-age hearing threshold was significant *β*  =  -0.163, *p* <0.001 and indicated that a higher cognitive ability in childhood was associated with better hearing in middle age. The cross-lagged effect from childhood hearing threshold to middle-age cognitive ability was smaller but also significant *β*  =  -0.076, *p*  =  0.001 and indicated that better hearing in childhood was associated with a higher cognitive ability in middle age.

**Figure 6.**
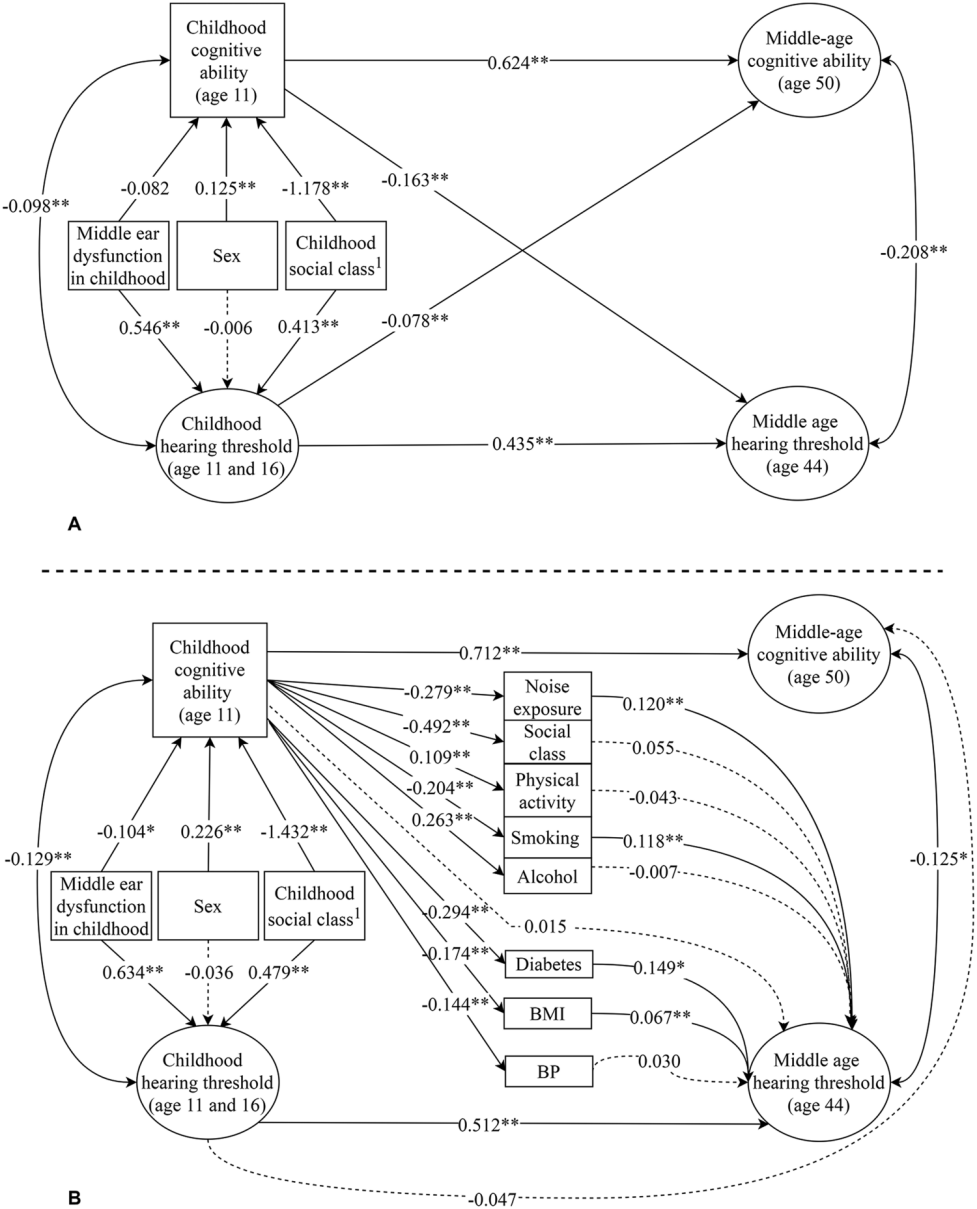
*Standardized parameter estimates from model 2 (panel A) and model 3 (panel B)*. *Note*. Double headed arrows represent correlations and single headed arrows represent regression effects. * indicates p < .05. ** indicates p < .01. Arrows with dashed lines are non-significant. ^a^Regression paths from childhood social class represent the effect of “unskilled” relative to the “professional” occupational social class.

Model 2, which controlled for potentially confounding variables in childhood, also fit the data adequately CLI  =  0.962, TFI  =  0.950, RMSEA  =  0.039. Estimates from Model 2 are shown in Figure 6a and Supplementary Table 9. In childhood, having a father in the “professional” relative to a “manual” childhood occupational social class was associated with poorer hearing; having a father in the “professional” relative to any other childhood occupational social class was associated with higher childhood cognitive ability. Being female was associated with a higher childhood cognitive ability but was unrelated to childhood hearing. Middle ear dysfunction was related to poorer childhood hearing. The correlation in Model 2 between childhood hearing threshold and cognitive ability was only slightly reduced from *r*  =  -0.120, *p* <0.001 (in Model 1) to *r*  =  -0.098, FDR *p*  =  0.001. The remaining parameter estimates including the correlation between middle-age hearing threshold and cognitive ability were largely unchanged.

Finally, in Model 3 we tested whether the cross-lagged association between childhood cognitive ability and middle-age hearing threshold was mediated by a set of occupational, demographic, lifestyle and health factors assessed in middle age. This model had an adequate fit to the data CLI  =  0.962, TFI  =  0.954, RMSEA  =  0.040. Estimates from Model 3 are shown in Figure 6b and Supplementary Table 10. Childhood cognitive ability was significantly related to each of the potentially mediating variables assessed in middle age. Specifically, a higher childhood cognitive ability was related, at age 44, to a lower risk of diabetes, more frequent physical activity, greater alcohol consumption, a more professional occupation, less smoking, less occupational noise exposure, a lower BMI, and lower blood pressure. Potentially mediating variables that were also associated with poorer middle-age hearing included history of diabetes, more smoking, greater occupational noise exposure*,* and a higher BMI. Both cross-lagged effects, from childhood cognitive ability to middle-age hearing threshold and from childhood hearing threshold to middle-age cognitive ability were attenuated to non-significance in this model. The correlation between middle-age hearing threshold and cognitive ability was slightly reduced but remained significant *r*  =  -0.125, FDR *p*  =  0.024.

### Subsidiary Analysis

In subsidiary analysis we additionally tested whether the relationship between childhood hearing threshold and middle-age cognitive ability was mediated by any of the occupational, demographic, lifestyle or health variables. Results from this analysis are shown in Supplementary Table 1^1^. This model also provided adequate fit the data well: CFI  =  0.950, TLI  =  0.939, RMSEA  =  0.046. Less good hearing in childhood was significantly related, at age 44, to a higher risk of diabetes, less physical activity, less alcohol consumption, a less professional occupation, more smoking, and a higher BMI. The effect sizes for these associations were small (ranging between −0.122 and −0.038). All of the potentially mediating variables were associated with middle-age cognitive ability.

Secondly, the factor loading of processing speed on the middle-age general cognitive ability latent variable was low. To test for associations between the hearing threshold variables and processing speed we re-ran Model 1 replacing middle-age cognitive ability with the processing speed variable. Results from this analysis are shown in Supplementary Table 1^2^. Processing speed was not significantly associated with hearing threshold in childhood or middle age.

Finally, a large proportion of the original NCDS sample was excluded from the analytical sample due to missing data on the childhood cognitive ability or hearing variables. To test whether this approach had biased our results, we re-ran Model 1, including participants with missing data on the these variables (sample N  =  13,927), note that participants with no data on any of the childhood cognitive ability or hearing variables were still excluded (N = 4,517). Parameter estimates from analysis with this larger sample (displayed in Supplementary Table 1^3^) were very similar to those reported in the main results (estimates were mostly identical when rounded to one decimal place).

## Discussion

The present study set out to test for possible reciprocal dynamic relationships between hearing and cognitive abilities from childhood to middle age using data spanning 39 years of life. Our key findings can be summarised as follows: 1) Better hearing (indicated by a lower overall hearing threshold) was associated with a slightly higher cognitive ability; this association was apparent in cross-sectional results in childhood and in middle age. 2) Between-person differences in hearing and cognitive abilities were relatively stable from childhood to middle age. 3) There was a cross-lagged association between higher childhood cognitive ability to better middle-age hearing ability, and a smaller but still significant association between better childhood hearing ability and higher middle-age cognitive ability. 4) Potentially confounding variables (childhood social class, sex, and history of middle ear dysfunction) did not fully account for the relationship between hearing and cognitive abilities in childhood. 5) The relationship between childhood cognitive ability and middle-age hearing ability was statistically mediated by occupational and health variables in adulthood: occupational noise exposure, BMI, history of diabetes, and smoking status. 6) The relationship between hearing and cognitive abilities in middle age was not fully explained by childhood hearing and cognitive abilities 6.

In interpreting these results, it is important to note the large number of participants included in our study. With a large sample size, there is a high likelihood of obtaining significant *p-*values, even for very small effects (Sullivan & Feinn, 2012). Therefore, close attention should be paid to the magnitude (not just the statistical significance) of the associations discussed below.

The small but significant relationship between hearing and cognitive abilities in childhood and middle age confirms previous research with healthy children and adult participants. As described in the introduction, children and adults with a higher cognitive ability have been found to perform better on a range of auditory processing tasks including auditory processing speed, discrimination, and acuity (Deary et al., 1989b; Deary et al., 2004; Helmbold et al., 2006; McCrory & Cooper, 2005; Parker et al., 1999; Raz et al., 1987; Watson, 1991). It is notable that none of these studies tested for an association between cognitive ability and hearing threshold level, which was the novel approach applied here; this test of hearing sensitivity taps a stage of auditory perception that potentially relies on fewer “top down” cognitive resources than tests of processing speed, discrimination, or acuity (which typically require participants to discriminate between different auditory stimuli). Therefore, our results could indicate that cognitive ability is associated with even relatively simple tests of sensory perception.

The present study included mostly individuals with normal hearing. We excluded participants who were identified as deaf in adulthood or reported a history of maternal rubella or meningitis in childhood from the analytical sample. However, it remains possible that the association between hearing and cognitive abilities observed in our study was driven by the small proportion of participants with moderate or severe hearing loss in childhood. As described in the introduction, previous studies have documented associations between childhood hearing impairment and poorer performance on some cognitive ability tests (Conway et al., 2009; Kral et al., 2016). We further tested whether the correlation between childhood hearing and cognitive abilities could be explained by differences in childhood social class, sex, or history of middle ear dysfunction. Whereas each of these potentially confounding variables was related to childhood hearing and/or cognitive ability, they did not fully account for the association between these variables. This result suggests that other factors or developmental processes, not accounted for in our models, drive the association between auditory and cognitive function in childhood.

Confirming earlier findings from studies with middle-aged and older adults (Humes, 2015; Livingston et al., 2017; Loughrey et al., 2017; Moore et al., 2014; Schubert et al., 2017), we found that hearing and cognitive abilities were positively correlated in middle age. This finding lends further support to the suggestion that associations observed in older age may originate in middle age or even earlier. Extending this perspective in the present study, we hypothesised that the association between hearing and cognitive abilities, observed in middle age, might in fact originate in childhood. However, we found that the relationship between middle-age hearing and cognitive abilities was not fully accounted for by childhood hearing and cognitive abilities, suggesting that factors specific to adulthood might partially drive some of this later-life association. Schubert et al. (2017) for instance, suggest that this association may be indicative of early brain ageing. This result would be predicted by a “common cause” account of ageing whereby cognitive and sensory abilities become more closely related as age-related changes begin to impact both domains (Baltes & Lindenberger, 1997; Christensen et al., 2001).

We found that between-person differences in hearing and cognitive abilities were relatively stable from childhood to middle age. Whereas the stability of cognitive ability across the life course has been documented by others (Deary et al., 2000; Gow et al., 2011), less has been published on the stability of hearing abilities (Russ et al., 2018). Therefore, it is noteworthy that childhood hearing ability predicted adulthood hearing ability with a medium to large effect size (minimally-adjusted *β*  *=*  0.430, fully-adjusted *β*  *=*  0.512). However, in the present study, assessments of hearing abilities were not equivalent in childhood and middle age. Relative to assessments in childhood, middle-age hearing ability was assessed using a shorter audiogram with fewer frequencies. The true associations between childhood and middle-age hearing abilities may differ from those documented here; it is possible that repeated assessment with a more sensitive instrument would detect subtle changes in hearing abilities that were not captured in the present study.

General cognitive ability was also assessed with different types of tests in childhood and adulthood. This difference might have affected the observed associations between cognitive and hearing abilities. Specifically, indicators of general cognitive ability in middle age included two tests that are sensitive to cognitive ageing effects, namely verbal memory and processing speed. We cannot rule out that these cognitive ability domains were more strongly negatively associated with hearing loss in middle age and that a weaker association would be observed if an IQ-type test had been used.

In addition to tracking the correlation between hearing and cognitive abilities from childhood to middle age, we tested for potential cross-lagged effects. The positive association between childhood cognitive ability and middle-age hearing ability observed here corroborates findings from our previous study with older adults, in which a higher childhood cognitive ability was related to a lower risk of hearing impairment in older age (Okely et al., 2019). However, in contrast with that study, in the present analysis childhood cognitive ability did not fully account for the relationship between middle-age hearing and cognitive abilities. Childhood cognitive ability is related to important health outcomes and health behaviours in adulthood (Batty et al., 2007; Singh-Manoux et al., 2009; Wraw et al., 2015; Wraw et al., 2018). This effect was apparent in the present study with a higher childhood cognitive ability predicting greater physical activity, less smoking, a lower risk of diabetes, a lower BMI and lower blood pressure in adulthood. These relationships have been documented previously in other samples from the UK and the USA (Batty et al., 2007; Wraw et al., 2015, 2018). A novel finding in our study was that a higher childhood cognitive ability was associated with less exposure to occupational noise in adulthood. It is likely that this association is a consequence of the link between higher cognitive ability and having a more professional occupation, which typically involves lower levels of occupational noise (Lie et al., 2016). Whereas childhood cognitive ability was related to all of the potentially mediating variables, only occupational noise exposure, smoking status, history of diabetes, and BMI were associated, negatively with hearing ability in middle age, making it possible that some or all of these variables play a role in mediating the relationship between childhood cognitive ability and middle-age hearing ability. However, it is also possible that our results reflect a non-causal effect. For instance, if childhood cognitive ability is independently associated with middle-age hearing ability and the potentially mediating variables, then these potentially mediating variables might act as “proxies” of cognitive ability (and account for variance in cognitive ability in the model) rather than causal or mechanistic mediators of the association between cognitive and hearing abilities.

We also observed a weak (*β*  =  -0.076) association between better childhood hearing ability and higher middle-age cognitive ability. This finding could indicate that any influence of hearing loss on cognitive development (Conway et al., 2009; Fitzpatrick, 2015; Kral et al., 2016) extends beyond childhood. We observed a weak but statistically significant association between childhood hearing threshold and most of the potentially mediating variables such that more sensitive hearing was related to better health, health behaviours and a more professional occupation in adulthood. It is possible that these relationships are mediated by cognitive ability or educational attainment in adolescence or confounded by other underlying health conditions.

The focus of the present study was on the relationship between hearing and cognitive abilities. However, this work sits in the wider context of research on cognitive abilities and sensory functions generally, including vision and touch (Humes, 2015). Perception via these sensory modalities, particularly vision, is also positively correlated with cognitive performance in children (C. S. Watson et al., 2003) and adults (Dupuis et al., 2015; Elyashiv et al., 2014), and the strength of association between sensory functions and cognitive performance increases when multiple senses are examined simultaneously (Humes, 2015). Future studies could apply the life course approach developed here with cognitive and hearing abilities, to study the interplay between cognitive ability and multiple senses over time.

The strengths of our study include the large sample of participants, the extensive follow-up period, and the range of potentially mediating and confounding variables that we could account for in our models. Limitations must also be considered. The audiogram tests in childhood (at ages 11 and 16) were conducted in locally available facilities and procedures were not standardised across sites (Fogelman, 1983). Therefore, it is likely that hearing threshold levels recorded in childhood less accurately reflect true hearing abilities than those recorded in adulthood, when standardised testing procedures were implemented by trained study research nurses (Ecob, 2008). This limitation may have increased the proportion of noise in the dataset and potentially resulted in a less accurate estimate of the associations between the hearing and cognitive ability variables. In addition, a large proportion of NCDS participants with missing data were excluded from the analytical sample. Excluded participants typically had higher hearing threshold levels and performed less well on most of the cognitive tests than participants who were included in the analytical sample. These differences were apparent in childhood and in adulthood. It is likely that excluding these participants resulted in an underestimate of the range of hearing and cognitive abilities in the general population, and potentially the strength of association between these variables. However, parameter estimates from subsidiary analysis including participants with missing childhood cognitive ability or hearing data were similar to those obtained with the original analytical sample (see subsidiary analysis and Supplementary Table 1^3^ for details). Previous work with the NCDS sample indicates that both myopia (short sightedness) and hearing ability may be impacted by early life development (Elliot & Vaitilingam, 2008). If visual function is correlated with hearing ability, it could act as a potential confound of the association between hearing ability and cognitive performance. However, in subsidiary analysis, we found that hearing threshold levels were not significantly higher among participants categorised as having a visual impairment. Furthermore, it should be noted that although the models described in this paper closely resemble cross-lagged panel models (Kearney, 2017), they diverge from this analytic approach in two important ways. Firstly, childhood and middle-age cognitive and hearing variables were not assessed by the same tests at each life stage; rather a different number or type of tests were used in childhood and middle age. Secondly, cognitive and hearing abilities within childhood and middle age were not assessed concurrently but were tested at different ages (11 and 16 in childhood, and 44 and 50 in middle age). Thus, the longitudinal associations between hearing and cognitive abilities reported here should be interpreted with caution and ideally replicated using data appropriate for cross-lagged panel analysis. Finally, most participants in our sample had normal hearing in childhood and adulthood with only a small proportion of participants showing signs of mild to severe hearing loss. Therefore, the findings documented here might not generalize to individuals with hearing loss and most likely reflect associations between hearing and cognitive abilities in the normal hearing population.

In summary, this study has for the first time demonstrated a life-long – from childhood to middle age – reciprocal and dynamic relationship between hearing and cognitive abilities. These new findings demonstrate the value of applying a life-course perspective to Cognitive Hearing Science research. Further, they open two new research topics: (1) studies with children could examine how associations between hearing and cognitive abilities, established at early stages of development, play out in adolescence and adulthood, and how these variables relate to other important life outcomes including physical health and health behaviours, and (2) research into hearing and cognitive abilities in older age should incorporate the potential contribution of pre-morbid hearing and cognitive abilities to this relationship.

## Supplemental Material

sj-docx-2-tia-10.1177_23312165211053707 - Supplemental material for Associations Between Hearing and Cognitive Abilities From Childhood to Middle Age: The National Child Development Study 1958 Click here for additional data file. Supplemental material, sj-docx-2-tia-10.1177_23312165211053707 for Associations Between Hearing and Cognitive Abilities From Childhood to Middle Age: The National Child Development Study 1958 by Judith A Okely, Michael A Akeroyd and Ian J Deary in Trends in Hearing
